# Collaborative reliance in medicine safety and quality regulation: Investigation of experiences in handling N-nitrosamine impurities among ZaZiBoNa participating countries

**DOI:** 10.3389/fmed.2022.975032

**Published:** 2022-09-09

**Authors:** Patience P. Shabangu, Rutendo J. Kuwana, Admire Dube

**Affiliations:** ^1^South African Health Products Regulatory Authority, Pharmaceutical Evaluations, Pretoria, South Africa; ^2^Incidents and Substandard/Falsified Medical Products Team, Regulation and Safety of Medicines Unit, World Health Organization, Geneva, Switzerland; ^3^School of Pharmacy, University of the Western Cape, Bellville, South Africa

**Keywords:** National Regulatory Authorities (NRAs) in Southern Africa, N-nitrosamine impurities, medicine quality and safety, reliance, Southern African Development Community (SADC), South African Health Products Regulatory Authority (SAHPRA), ZaZiBoNa

## Abstract

**Introduction:**

The presence of N-nitrosamine impurities in medicines raised concerns globally as they are genotoxic and probable human carcinogens. A review of N-nitrosamine impurities in medicines provides an opportunity for National Regulatory Authorities (NRAs) to ensure that corrective and preventive actions are applied so that safe and good quality medicines are made available to the public. This study aimed to investigate the experiences on reviews conducted by NRAs from various Southern African Development Community countries which participate in the regional work-sharing forum, ZaZiBoNa, on the quality and safety data due to the presence of N-nitrosamine impurities in medicines.

**Methods:**

A comparative, descriptive study using mixed methods was conducted. Purposive sampling was applied in selecting research participants based on their participation status in the ZaZiBoNa initiative. A standardized questionnaire structured into five parts was completed by ZaZiBoNa focal persons/nominated individuals to determine the experience of each NRA in addressing the safety and quality issues related to the presence of N-nitrosamine impurities in the affected medicines. Profiled medicines included sartans, ranitidine, metformin, rifampicin, and rifapentine.

**Results:**

Sartan medicines had been reviewed by all countries participating in the ZaZiBoNa initiative. Although most NRAs have yet to conduct reviews on other profiled medicines, evaluations have been implemented to ensure access to safe and good quality medicines within the region. Most countries experienced challenges in communicating with applicants or marketing authorization holders (MAHs) on reviewing N-nitrosamine impurities in their medicines. The majority of NRAs agree that there is a need for further collaboration efforts to review N-nitrosamine impurities in medicines.

**Conclusion:**

The review of N-nitrosamine impurities in the profiled medicines by NRAs within the region has demonstrated the importance of enhanced regulatory oversight to safeguard against the risks associated with medicines. Collaborative reliance on the review of the safety and quality of medicine, continuous monitoring, implementation and review of processes, testing methods, and regular engagements with stakeholders could be essential in ensuring adequate control of N-nitrosamine impurities in medicines.

## Introduction

The presence of N-nitrosamine impurities in medicines has raised global regulatory concerns. In 2018, the European Medicines Agency (EMA) and the United States Food and Drugs Administration (US FDA) initiated a review on valsartan-containing products after an active pharmaceutical ingredient (API) manufacturer detected an impurity N-nitrosodimethylamine (NDMA) during the manufacture of the API, valsartan (1, 2). As of 2019, it had been reported that N-nitrosamine impurities were detected in other medicines such as ranitidine which are indicated for the treatment and prevention of ulcers, and extended-release metformin formulations used for the treatment of diabetes mellitus (3–5).

By August 2020, certain samples of rifampin and rifapentine used to treat tuberculosis (TB) were reported to contain nitrosamine impurities (6, 7). In July 2021, it was found that varenicline, used to stop smoking, was affected by the presence of N-nitroso-varenicline above the acceptable limit (8, 9). No data has been published on the impurities’ presence in the African region. The presence of these impurities in selected medicines with or pending marketing authorization within the region should therefore be investigated.

N-nitrosamine impurities may be generated when secondary or tertiary amines react with nitrites under acidic conditions (10–2). Notable sources for the formation of nitrosamines include the API, intermediates, or starting materials processed under specific conditions and in the presence of certain reagents, solvents, raw materials, and processing aids (10). If the starting materials and raw materials are contaminated, nitrosamines may be formed. In certain cases, the API may undergo degradation pathways during storage that form nitrosamine impurities. In the manufacturing process, cross-contamination of the materials if the equipment used is not cleaned adequately could lead to nitrosamine formation, together with the use of certain packaging materials for the final pharmaceutical products which may also result in the formation of nitrosamine impurities. The World Health Organization (WHO) Prequalification of Medical Products unit (PQT/MED), EMA and USFDA amongst other medicine regulators have requested applicants or marketing authorization holders (MAHs) to test the nitrosamine impurity in a representative number of batches for the API and the final pharmaceutical product (FPP) to evaluate the risks associated with nitrosamine impurities (2, 11, 13).

Different approaches are applied for setting the limits of impurities. Among other regulators, the EMA, USFDA, and WHO have published the interim allowable daily intake (AI) limits for the impurities as per ICH-M7 guidelines on the assessment and control of DNA reactive (mutagenic) impurities in pharmaceuticals to limit potential carcinogenic risk (4, 11, 12). Another approach that can be applied on a case-by-case basis, especially for medicines used short term, is the less-than-lifetime (LTL) approach, which allows for an acceptance of much higher levels of nitrosamine impurities (14).

For new nitrosamines, the acceptable limits may be set by extrapolation from available animal data. If there is no adequate animal data, the threshold of toxicological concern (TTC) of 18 ng/day for nitrosamines can be used (14).

The USFDA, EMA, Health Canada, and other regulatory authorities have published different test methods, which are recommended to be used for the determination of N-nitrosamine impurities in the affected products, such as; the gas chromatography-mass spectrometry (GC/MS) headspace method for sartan medicines, Liquid Chromatography-High Resolution Mass Spectrometry (LC-HRMS) Method for the determination of NDMA in Ranitidine, Liquid Chromatography-Tandem Mass Spectrometry (LC-MS/MS) Method for the Determination of NDMA in Ranitidine amongst others (15). These are complex methods using expensive equipment. Qian et al. demonstrated that in 52% of 69 batches of tested metformin extended-release formulations and 17% of 59 tested batches of immediate release, samples contained NDMA impurities above acceptable limits (5). The selected method for the determination of nitrosamine impurities in medicines should be shown to be suitable for the product under review (16).

Table 1 lists the interim allowable daily intake limits for N-nitrosamine impurities in medicinal products by the EMA, USFDA, and WHO.

**TABLE 1:** Interim acceptable daily intake limits published for N-nitrosamine impurities in medicinal products (4, 11, 12).

N-nitrosamine impurity chemical name (abbreviated)	Acceptable limits ng/day		
	
	EMA	USFDA	WHO
N-Nitrosodimethylamine (NDMA)	96.0	96	96.0
N-Nitrosodiethylamine (NDEA)	26.5	26.5	26.5
N-Nitrosoethylisopropylamine (EIPNA)	26.5	26.5	26.5
N-Nitrosodiisopropylamine (DIPNA/NDIPA)	26.5	26.5	26.5
N-Nitroso-N-methyl-4-aminobutyric acid (NMBA)	96.0	96	96.0
1-Methyl-4-nitrosopiperazine (MeNP)	26.5		
N-Nitroso-di-n-butylamine (NDBA)	26.5		
N-Nitroso-N-methylaniline (NMPA)	34.3	26.5	
N-nitrosomorpholine (NMOR)	127		
N-nitroso-varenicline (NN)	37.0		

Regulatory authorities are constantly monitoring medicines to ensure that the available products on the market have been tested and are within the acceptance criteria. New developments are regularly reported on the presence of nitrosamines in medicines. For those medicines that are pending marketing authorization, they are monitored to ensure adequate control for the impurities in the medicinal product applied for is well characterized in the respective dossier. Since the initial detection of N-nitrosamine impurities, recalls of products have been initiated either by manufacturers or directed by NRAs. The presence of nitrosamine impurities may also affect medicine shortages due to imposed embargos whilst reviews are conducted.

The ZaZiBoNa collaborative medicines registration initiative was established in 2013 to facilitate work-sharing between NRAs in SADC to reduce duplication of efforts and facilitate access to medical products for the public (17). Countries actively participating in ZaZiBoNa include Zambia, Zimbabwe, Botswana, Namibia, South Africa, Democratic Republic of Congo, Tanzania, Malawi, and Mozambique, with other countries, namely; Angola, Seychelles, Swaziland, and Madagascar, participating as non-active members/observers (17). Countries with active member status, are those that have legislation and guidelines on the registration of medicines and in-house capacity to perform assessments of registration dossiers or good manufacturing practice (GMP) inspections (17).

Regulatory reliance is defined by the WHO as the act whereby national regulatory authorities (NRAs) in one jurisdiction may take into account and rely upon evaluations performed by another NRA or trusted institution in reaching its decision (18). For example, reliance allows low to middle income NRAs with limited capacity to rely on well-resourced mature recognized regulatory authorities (RRAs) such as EMA, FDA, and others, for decisions to fulfill the goal of protecting and promoting public health by ensuring that all medicines are of good quality, safety, and efficacy (19). Since March 2019, NRAs participating in the ZaZiBoNa initiative reviewed sartan medicines to ensure that the limits of the impurities are within the acceptable proposed limits. Recommendations drawn from EMA’s approach to the list of outstanding issues to be addressed by the active substance manufacturers for angiotensin-II receptor antagonists (sartans) were jointly reviewed and adapted for ZaZiBoNa (20). NRAs were required to send out communications with requests to their respective applicants/MAHs to review the presence of N-nitrosamine impurities. Each NRA was to set timelines for applicants to respond to the queries/recommendations raised.

This study aimed to investigate the experiences on reviews conducted by NRAs which participate in ZaZiBoNa on the quality and safety regulation of N-nitrosamine impurities in medicines. It describes the procedures followed and compares data submitted within the NRAs in ensuring adequate control of N-nitrosamine impurities in medicines. South African Health Products Regulatory Authority (SAHPRA) was investigated further, in relation to how it navigated this complex situation. SAHPRA applied reliance in the review of N-nitrosamine impurities by leveraging on data published by the EMA, USFDA, WHO, and other regulators with which it bilaterally or unilaterally aligns.

## Materials and methods

### Study design

A comparative, descriptive research design using mixed methods was employed to meet the objectives of the study.

### Study participants

Purposive sampling was used in the selection of research participants. Nine ZaZiBoNa focal persons or nominated individuals from the countries actively participating in the ZaZiBoNa initiative were requested to participate in the study through email between October and November 2021. Ethics approval was obtained from the University of the Western Cape Biomedical Research Ethics Committee (Ethics Reference Number: BM21/9/2). The countries invited to participate in the study were NRAs from Botswana (Botswana Medicines Regulatory Authority—BOMRA), Democratic Republic of Congo (Agence Congolaise de Réglémentation Pharmaceutique—ACOREP), Malawi (Pharmacy and Medicines Regulatory Authority—PMRA), Mozambique (National Directorate of Pharmacy—DNF), Namibia (Namibia Medicines Regulatory Council—NMRC), South Africa (SAHPRA), Tanzania (Tanzania Medicines and Medical Devices Authority—TMDA), Zambia (Zambia Medicines Regulatory Authority—ZAMRA) and Zimbabwe (Medicines Control Authority of Zimbabwe—MCAZ).

### Data collection

An online questionnaire was generated and distributed using QualtricsXM. The researchers designed the questionnaire to capture data on the experiences of the NRAs actively participating in the ZaZiBoNa initiative in handling the presence of N-nitrosamine impurities in medicinal products. The questionnaire was structured in five parts based on a review of literature which included publications such as journals, web searches, and regulatory guidances/guidelines.

Part A focused on identifying the represented NRA and outlining publication purposes. Part B specified the profiles of medicines affected by the presence of N-nitrosamine impurities as investigated by the EMA and USFDA, namely; sartan medicines (containing either azilsartan, candesartan, eprosartan, irbesartan, losartan, olmesartan, telmisartan, or valsartan); ranitidine medicines; metformin medicines; rifampicin medicines and rifapentine medicines and reviewed data on the number of products that are registered or pending registration within the NRAs. Part C highlighted regulatory activities followed by NRAs to ensure adequate control of the N-nitrosamine impurities in medicines. Part D covered data to determine the participants’ knowledge of laboratory practices by NRAs to test for the N-nitrosamine impurities. Part E reviewed challenges experienced and possible solutions suggested by NRAs in addressing the regulation of N-nitrosamine impurities.

The structured questionnaire was developed using data obtained following a pilot study to determine the feasibility of the research topic. The questionnaire was validated through a pilot by six representatives from the NRAs participating in ZaZiBoNa. The same NRAs were also included in the final study to determine the experiences that emerged from the medicine regulators within the region to ensure that the safety and quality issues related to carcinogenic N-nitrosamine impurities are monitored. The questionnaire was then amended and improved by the researchers following the pilot in order to accomplish the objectives of the study.

### Data analysis

Data analysis was performed for all affected products on registered applications and applications in the registration pipeline from the seven countries actively participating in the ZaZiBoNa initiative. Content was reviewed and analyzed on the handling of N-nitrosamine impurities by NRAs in the identified medicines.

## Results

Seven out of 9 (78%), focal persons/nominated individuals from the NRAs completed the questionnaire and data was analyzed.

### Medicinal products profile

**FIGURE 1:**
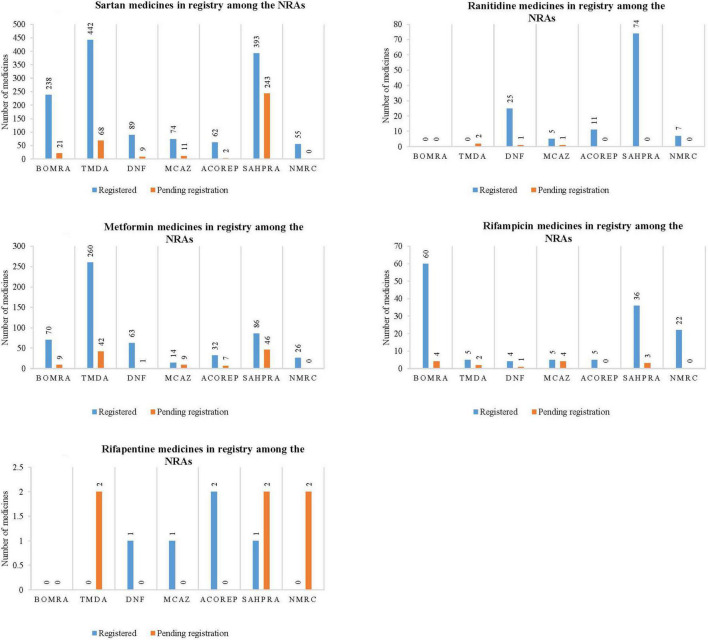
Graphical representation of the number of medicines that are registered and pending registration within the national regulatory authorities for sartan medicines, ranitidine medicines, metformin medicines, rifampicin medicines, and rifapentine medicines.

The study reviewed data on the profiled medicines that are registered and pending registration within the NRAs participating in the ZaZiBoNa initiative, as represented in Figure 1. The majority of NRAs have conducted reviews on the profiled medicines.

Sartan medicines represented the majority of medicine applications reviewed by all seven NRAs, as noted in Figure 1.1. TMDA had the highest number of sartan medicines that are registered (442), followed by SAHPRA (393), BOMRA (238), DNF (89), MCAZ (74), ACOREP (62), and lastly NMRC (55). SAHPRA had the highest number of sartan medicines pending registration (243), with ACOREP representing the lowest (2). In total, 1,353 sartan medicines have been registered, while 354 are pending registration within the NRAs.

Most NRAs indicated that there are ranitidine medicines, while 2 out of 7 NRAs (29) indicated that there are no ranitidine medicines within their NRA register. SAHPRA had the highest number of registered ranitidine medicines when compared to MCAZ, which had the least number (5) (Figure 1.2). Although two regulatory authorities, BOMRA and TDMA did not have registered ranitidine medicines, the number of applications received for ranitidine marketing authorizations by other NRAs was significant. In total, 122 ranitidine medicines have been registered, while four are pending registration amongst the NRAs.

All NRAs had registered metformin medicines, with TDMA having a large number of medicines (260) compared to other regulatory authorities, which had the least number of applications. A total of 551 metformin medicine have been registered, while 114 are pending registration (Figure 1.3) across all NRAs.

Figures 1.4, 1.5 demonstrate that while rifampicin and rifapentine are both used in the treatment of tuberculosis, most NRAs participating at ZaZiBoNa had reviewed more rifampicin medicines’ applications when compared to rifapentine. This may be attributed to the approval timelines as rifampicin was granted marketing authorization by the USFDA in 1971 while rifapentine was initially approved in 1998 and generally used in TB multi-drug resistant prevalence settings. In total, 135 rifampicin containing medicines have been registered, while 14 are pending registration and five rifapentine containing medicines have been registered, while six are pending registration within the NRAs.

**FIGURE 2:**
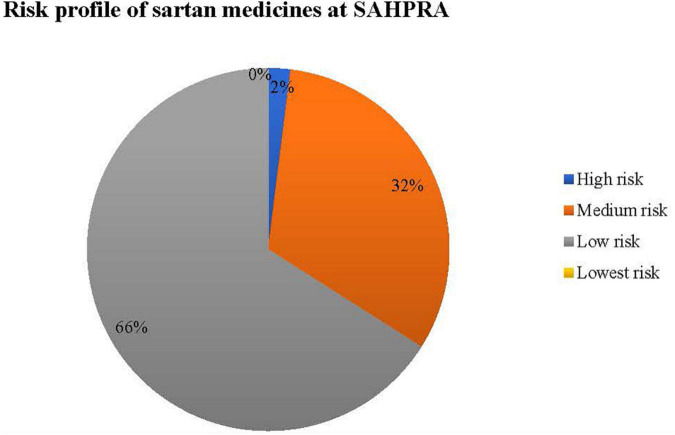
Risk matrix profile for N-nitrosamine impurities in sartan medicines for SAHPRA.

Sartan medicines for submissions received at SAHPRA were risk profiled for N-nitrosamine impurities as per Figure 2.

Table 2 provides the criteria used to classify risk as applied in the sartan medicines applications received at SAHPRA. High risk sartan medicines were those which were initially reported by EMA and USFDA following the detection of N-nitrosamine impurities by the API manufacturers, in which the APIs, valsartan and losartan were affected. All other valsartan and losartan medicines submitted for market authorization at SAHPRA were classified as medium risk. Applications for sartan medicines containing either azilsartan, candesartan, eprosartan, irbesartan, olmesartan, or telmisartan, in which no certificate of suitability (CEP) as issued by the European Directorate for the Quality of Medicines and HealthCare was submitted were classified as low risk. Sartan medicines in which a CEP was submitted in support of the application for registration of medicines were regarded as lowest risk. 66% of the sartan medicine applications submitted at SAHPRA were of the lowest risk from the formation of N-nitrosamine impurities. To assess the risk of nitrosamine impurities in the sartan medicines, a risk matrix was designed for the submissions received at SAHPRA (Figure 2).

**TABLE 2:** Risk classification profile for N-nitrosamine impurities in sartan medicines for SAHPRA.

Risk matrix profile	Reason
High risk	EMA reported API manufacturer and FDA reported API manufacturer
Medium risk	All other valsartan and losartan containing products
Low risk	All other sartan containing medicines
Lowest risk	All sartan medicines in which a Certificate of Suitability (CEP) has been issued

Data presented is for sartan medicines manufactured either as a single molecule or in combination with other APIs.

### Regulatory responses and decisions

All NRAs 100% (*n* = 7) had at least conducted a review for the presence of nitrosamines on sartan medicines in their registers. Although 7 NRAs participated in the study, only 6 NRAs provided a response on the medicines reviewed by the NRAs. Amongst the represented NRAs, 83% (5 out of 6) had conducted reviews on the investigation of nitrosamine impurities in ranitidine medicines, while 67% (4 out of 6) reviewed metformin medicines; 17 (1 out of 6) rifampicin medicines, and 33% (2 out of 6) reviewed rifapentine medicines. Respondents indicated that there was also poor follow up by the NRAs with the applicants or market authorization holders due to resource constraints within the NRAs.

Six out of 7 NRAs had communicated with applicants on the review of nitrosamines in the affected medicines. The timeframe for applicants to respond to the queries raised was 6 months for most NRAs. However, some authorities like SAHPRA had extended the response time further to an additional 6 months. In all NRAs, the limits of nitrosamine impurities in the responses reviewed for medicine applications were within the acceptance criteria as set by EMA, USFDA, and WHO. Four authorities, namely; BOMRA, SAHPRA, TDMA, and MCAZ, received voluntary suspensions or withdrawal of applications for registration of medicines by applicants following the communication on the review of nitrosamines in medicines. The NRAs initiated recalls, suspensions, or cancelation of applications as measures to control the nitrosamine impurities in the affected medicines in line with reviews conducted by EMA and the USFDA.

SAHPRA operations are undertaken by different units/departments. Within registry, a total of 26 sartan medicines were voluntarily withdrawn by applicants while the following products have been recalled by the Regulatory Compliance unit pending outcomes of the investigations, sartan medicines (5); metformin medicines (1) and unspecified number of ranitidine medicines. Data on the number of withdrawals, suspensions or recalls from the other NRAs was not collected as some NRAs did not have resources to follow up on applicants.

### Access to testing laboratories

The majority of NRAs 86 (6 out of 7) had not requested for their laboratory to test for the N-nitrosamine impurities in medicines. Three NRAs, namely; MCAZ, SAHPRA, and TDMA, indicated that they had accredited laboratories within the regulatory authority. There are plans for testing for the presence of N-nitrosamine impurities if required by DNF, MCAZ, TDMA, and SAHPRA. However, most laboratories 60% (3 out of 5) do not have access to testing methods, equipment, and trained analysts to test for N-nitrosamine impurities in medicines. All respondents were not aware of any proposals for regional collaboration in conducting tests for the N-nitrosamine impurities in medicines.

### Regulatory experience

The challenges and possible solutions suggested by NRAs within the ZaZiBoNa participating countries during the investigation of N-nitrosamine impurities in medicines are summarized in Table 3. Furthermore, information is presented to address the need for further collaboration efforts to assess N-nitrosamine impurities in medicinal products.

**TABLE 3:** Challenges and possible solutions to the NRAs in the investigation of N-nitrosamine impurities.

Challenges experienced by NRAs during the investigation of N-nitrosamine impurities	Possible remedies to challenges proposed by NRAs
Applicants took time to respond and address Nitrosamine impurity concerns, resulting in some applications not being sufficiently followed up.	• Create logs with all affected products and follow up with applicants who have not fully addressed this concern.
Low/no responses received by NRAs from applicants	• Resend letters to the applicant/market authorization holder and set deadlines for submission of requisite data. • Arrange meetings with all stakeholders (applicants, NRA staff, and laboratory staff) to address the challenges faced and to guide applicants on the requirements. • Identify products that are undergoing renewals and review for the presence of Nitrosamine impurities.
There is poor communication with applicants.	• Improve communication with applicants. • Regular follow up and enforcement should be enhanced.
Tests for N-nitrosamine impurities use methods that are non-routine for the authority, for example, GC-MS/MS.	• Authority may opt to use HPLC-UV methods in the event that there might be requests to carry out testing of medicinal products.
Applicants requested to use the TTC limits instead of those proposed by EDQM and USFDA.	• No proposed solution as this may require further country or region-specific considerations.

Four out of 7 NRAs strongly agree, and three out of 7 NRAs agree that there is a need for further regional collaboration efforts on the assessment of N-nitrosamine impurities in medicines.

## Discussion

This study investigated the experiences of 7 NRAs that are actively participating in the ZaZiBoNa initiative to the quality and safety regulation of N-nitrosamine impurities in medicines. The EMA and USFDA have published data on the products affected by N-nitrosamine impurities, namely; sartan medicines (containing either azilsartan, candesartan, eprosartan, irbesartan, losartan, olmesartan, telmisartan, or valsartan); ranitidine medicines; metformin medicines; rifampicin medicines and rifapentine medicines (9, 21).

There is a large number of applications submitted for registration by applicants or market authorization holders within the region for the profiled medicines. This is overwhelming for NRAs. Although reviews have not been conducted in all profiled medicines by all NRAs, there are systems in place within the NRAs to ensure adequate control of the nitrosamine impurities in medicines applied for within the region. Further interventions should be undertaken by NRAs in order to protect the public within the region from consuming medicines that are affected by these impurities. The limits of the impurities were found to be within the interim acceptable limits. Noting the number of medicines recalled at SAHPRA, NRAs should develop electronic systems within the respective units/departments to ensure consistency in data reporting. It is recommended that in instances where the applicant or market authorization holder has opted for using the TTC limits instead of the proposed limits, the onus should be upon the applicant to demonstrate equivalency or superiority over the preferred limits with scientific justification.

Only three out of 7 respondents NRAs have accredited laboratories. However, they did not have the competency to test for N-nitrosamines. Capacity building is necessary for NRAs in low to middle-income countries to be able to test for the nitrosamines in medicinal products so that there is access to safe and good quality medicines available within the SADC region. Recent events point to the fact that more chemical groups could potentially be at risk of similar contamination with N-nitrosamines. This will present more pressure to the incapacitated NRAs. Improvements in access to testing methods, equipment, and trained analysts to test for N-nitrosamine impurities in medicines should be implemented as only a few NRAs have access. For this reason, NRAs should have access to laboratories meeting credible international norms and standards, should be adequately resourced with the required trained and competent staff, equipment, processes, methods, and apply effective quality management systems to test for the impurities in medicinal products. Having an effective laboratory quality management system is essential in ensuring that the quality assurance and quality control measures are built into the process of reviewing N nitrosamine impurities.

Most challenges raised were related to effective communication channels and follow up. There should be improvements in engagements between NRAs and stakeholders to address the quality and safety issues related to nitrosamines in medicines. Communication with manufacturers through establishing quality systems with agreed key performance indicators should be improved. The low response rate from applicants to the queries/recommendations sent was raised as a challenge by most NRAs, and this could be attributed to COVID-19 as there were disruptions in the work activities. Some activities completed within the NRAs were based on paper-based reviews and physical submission of the responses by applicants e.g., SAHPRA was still accepting responses printed on paper or by CD. Applicants were required to go directly to the authority to submit the responses or to upload their documents onto the IT system. Working conditions had to be changed and review processes amended. As NRAs are adjusting to the new way of working, follow-ups should be implemented by NRAs to ensure that the challenges related to the control of nitrosamine impurities in medicines are addressed rapidly. NRAs should be encouraged to collect and publish data on the number of recalls, suspensions and withdrawals as this will promote transparency within regulators.

Risk profiling of products at SAHPRA could only be completed on sartan medicines. The other medicines were not profiled due to the low response rate. This low response could be attributed to COVID-19, which affected the NRA and applicants/MAHs as the working conditions changed and regulatory processes had to be amended. Further follow-ups on the queries raised should be made with the relevant stakeholders to ensure that the limits of impurities in the applications submitted at SAHPRA are reviewed and confirmed for quality and safety.

The information gained since the initial detection of nitrosamines has contributed significantly to the new developments in testing methods and on setting the limits of impurities. Regulatory authorities such as EMA and FDA have published information on the nitrosamines; generated guidelines for setting the specification limits for the nitrosamines; recommendations for applicants and manufacturers in ensuring adequate control of the nitrosamine impurities; developed the requirements for testing and analytical methods; published steps to be taken during the review of nitrosamines and templates to be completed by applicants. Furthermore, EMA has published a document on the lessons learned from the presence of N-nitrosamine impurities in sartan medicines, which addresses the challenges, developments, and opportunities for a way forward in the review and control nitrosamines (22). Although more work still needs to be done as new developments are emerging, NRAs within the region could rely or recognize the work that has already been conducted by other regulatory authorities to reduce the regulatory workload.

Advocacy webinars hosted by representatives of the regulatory authorities intended for industry personnel and publications to patients and healthcare professionals are key regulatory communication tools. These activities promote transparency in the system and publishing data acts as a tool for regulatory authorities to build and establish confidence in their review processes and assure medicines’ safety and quality (23). NRAs within the region could implement such processes to promote trust in the regulatory activities undertaken by NRAs and improve compliance in the administration of medicines available in the market by consumers within the region.

Due to the lack of capacity within NRAs in the region, collaborative reliance on the regulation of the safety and quality of medicine has the potential to contribute positively to the improvements in the efficiency of the regulatory systems to ensure adequate control of the N-nitrosamine impurities in medicines. NRAs should develop and implement processes and testing methods that would be applied in testing for the presence of nitrosamine impurities. This would be essential to assure the quality and safety of the medicines accessed in the region.

Regulatory processes, guidelines, and policies may require updates to include measures on the handling of nitrosamine impurities in medicinal products. The EMA has published data on the lessons learned from the presence of N-nitrosamine impurities in sartan medicines. Key lessons learned may be drawn by continually monitoring, engaging with stakeholders, and implementing and reviewing processes related to the nitrosamine impurities in medicines within the region. This would benefit not only the NRAs but all relevant stakeholders as the safety of medicines within the region would be assured. Trust in the regulatory processes would be promoted, and there would be improvements in compliance in the administration of medicines by consumers within the region.

### Recommendations

The study identified the challenges and possible solutions to controlling N-nitrosamine impurities in medicines. NRAs should allocate more resources on the review of N-nitrosamine impurities as there seems to be more burden with more products being affected. It is suggested that a regional collaborative initiative be implemented in the evaluation of nitrosamine impurities to reduce the regulatory workload incorporated into the ZaZiBoNa workplan. As there are limited resources, expertise and capacity to apply time-sensitive compliance, responsibilities to evaluate risk should be shared amongst ZaZiBoNa participating countries. The presence of N-nitrosamines impurities is a global regulatory concern and there is therefore a need for increased reliance and harmonization of procedures in the review of N-nitrosamines by regulatory authorities to ensure access to safe, effective, and good quality medicines. As part of reliance, NRAs particularly from low-middle income countries, could review their regulatory policies to allow for the decisions taken by other regulatory authorities to be applied in their reviews to reduce duplication of review efforts. The review through reliance for the N-nitrosamine impurities could be a catalyst as most authorities are keen to use the reliance approach.

Frequent training of assessors and laboratory personnel to evaluate the presence of nitrosamine impurities should be conducted. NRAs should collaborate with the laboratory staff and develop processes and testing methods for testing the N-nitrosamine impurities. Pooling of resources to capacitate a few regional laboratories may be necessary.

Collaboration on pharmacovigilance activities within the ZaZiBoNa initiative could be established to promote access to safe and high-quality products within the region. NRAs within the region should establish collaborative pharmacovigilance and post-marketing surveillance programs, as implemented by, e.g., USFDA Medwatch or EMA EudraVigilance for updates on the safety and quality of medicines to promote transparency within NRAs. Patient awareness of the adverse event reporting system within the NRAs should be promoted.

NRAs and stakeholders (applicants, API, and FPP manufacturers) should ensure that corrective and preventive actions to prevent or reduce the formation of N-nitrosamine impurities during the manufacture of medicines are in place. Self-regulation should as much as possible be promoted.

### Study limitation

The study did not compare data to determine similarities within the applications submitted for registration among the NRAs. Future studies could be conducted on such reviews to determine how resources can be used effectively to evaluate the presence of nitrosamine impurities in medicines. Reliance from other agencies may be applied if the data submitted in the applications for market authorizations has been confirmed to be the same. Although the study did not capture the number of medicines withdrawn, suspended or recalled from the other NRAs, it would be suggested, that such data be captured in order to determine the impact of the suspensions, withdrawals and recalls on access to medicines.

## Conclusion

The study reviewed the responses of various countries in the region to control nitrosamines in medicines. Although the detection of nitrosamine impurities in medicines pose a serious challenge to the safety and quality of medicines, NRAs within the region are making concerted but severely limited efforts within their resource settings to ensure that there is adequate control of the limits of these impurities in medicines. This is in order that only medicines that are safe and of good quality are made available on the market. Opportunities for collaborative efforts in the review of the nitrosamines impurities in medicines could assist in reducing the regulatory workloads within NRAs.

## Data availability statement

The original contributions presented in this study are included in the article/supplementary material, further inquiries can be directed to the corresponding authors.

## Ethics statement

The studies involving human participants were reviewed and approved by the University of the Western Cape Biomedical Research Ethics Committee (Ethics Reference Number: BM21/9/2). The patients/participants provided their written informed consent to participate in this study.

## Author contributions

PS, RK, and AD contributed substantially to the conception and design of the study, contributed to the analysis and interpretation of data for the study, and read and approved the final manuscript. PS collected the data and wrote the first draft of the manuscript. All authors critically reviewed and revised the work.

## Abbreviations

ACOREP: Agence Congolaise de Réglémentation Pharmaceutique
AI: Allowable Daily Intake
API: Active Pharmaceutical Ingredient
BOMRA: Botswana Medicines Regulatory Authority
CEP: Certificate of Suitability
DNF: National Directorate of Pharmacy
EMA: European Medicines Agency
FPP: final pharmaceutical product
GC/MS: Gas Chromatography-Mass Spectrometry
GMP: Good Manufacturing Practice
LC-MS/MS: Liquid Chromatography-Tandem Mass Spectrometry
LC-HRMS: Liquid Chromatography-High Resolution Mass Spectrometry
LTL: Less-Than-Lifetime
MAHs: Marketing Authorization Holders
MCAZ: Medicines Control Authority of Zimbabwe
NDMA: N-nitrosodimethylamine
ng/day: nanograms per day
NMRC: Namibia Medicines Regulatory Council
NRAs: National Regulatory Authorities
PMRA: Pharmacy and Medicines Regulatory Authority
PQT/MED: Prequalification of Medical Products
RRAs: Recognized Regulatory Authorities
SADC: Southern African Development Community
SAHPRA: South African Health Products Regulatory Authority
TB: Tuberculosis
TMDA: Tanzania Medicines and Medical Devices Authority
TTC: Threshold of Toxicological Concern
US FDA: United States Food and Drugs Administration
WHO: World Health Organization
ZAMRA: Zambia Medicines Regulatory Authority.
